# Effect of renin angiotensin system inhibitors on long-term major cardiovascular outcomes in patients with high atherosclerotic cardiovascular risk

**DOI:** 10.1038/s41598-023-50430-8

**Published:** 2023-12-27

**Authors:** Wanwarang Wongcharoen, Nichanan Osataphan, Siriluck Gunaparn, Suphot Srimahachota, Pornwalee Porapakkham, Somchai Dutsadeevettakul, Arintaya Phrommintikul

**Affiliations:** 1 Department of Internal Medicine, Faculty of Medicine, Chiang Mai University, 110 Intawaroros Rd., Sriphoom, Muang Chiang Mai, 50200 Thailand; 2 Department of Internal Medicine, Faculty of Medicine, Chulalongkorn University, Bangkok, Thailand; 3 Central Chest Institute of Thailand, Nonthaburi, Thailand; 4 Department of Medicine, Golden Jubilee Medical Center, Mahidol University, Nakhon Pathom, Thailand

**Keywords:** Cardiology, Health care

## Abstract

The advantage of angiotensin converting enzyme inhibitor (ACEI) and angiotensin receptor blocker (ARB) in patients with preserved LV systolic function is uncertain. We aimed to investigate the effects of ACEI/ARB in high atherosclerotic risk patients without overt heart failure (HF) on long-term major cardiovascular outcomes (MACEs). The Cohort Of patients with high Risk for cardiovascular Events (CORE-Thailand) registry is a prospective, multicenter, observational, longitudinal study of Thai patients with high atherosclerotic risk. The patients with ejection fraction < 50% were excluded. Among 8513 recruited patients, there were 4246 patients included into final analysis after propensity score matching. At 5-years follow-up, Cox regression analysis showed that ACEI/ARB was significantly associated with reduced risk of all-cause mortality or non-fatal myocardial infarction, non-fatal stroke and HF hospitalization (HR 0.82, 95% CI 0.70–0.96, *P* = 0.011). The benefit was driven by the reduced all-cause mortality and HF. Subgroup analysis demonstrated that ACEI/ARB decreased risk of long-term MACEs in patients with diabetes (HR 0.77, 95% CI 0.63–0.94, *P* = 0.011) and patients not taking statin (HR 0.57, 95% CI 0.40–0.82, *P* = 0.002). We demonstrated that the use of ACEI/ARB was associated with reduced risk of long-term MACEs in a large cohort of high atherosclerotic risk patients. Reduction of all-cause mortality and HF were likely the main contributors to the benefit of ACEI/ARB.

## Introduction

The benefit of angiotensin converting enzyme inhibitor (ACEI) or angiotensin receptor blocker (ARB) has been established in patients with left ventricular (LV) systolic dysfunction^1^. It is well-described that the activation of renin angiotensin system (RAS) also has detrimental effects in other cardiovascular diseases including hypertension and coronary atherosclerosis^2,3^. The primary RAS effector, angiotensin II, has direct effects on renal sodium homeostasis and vasomotor tone, which are fundamental for blood pressure regulation and cardiovascular homeostasis. Angiotensin II also exerts strong mitogenic, pro-inflammatory, and pro-fibrosis actions through a variety of receptors^4^. Through the control of two crucial processes, inflammation and fibrosis, the RAS, and particularly Angiotensin II, contributes to the development of atherosclerosis^5^. Therefore, it is possible that the RAS blocking effects of ACEI/ARB would have potential therapeutic benefit in individuals with high atherosclerotic risk irrespective of heart failure.

However, there are conflicting data regarding the advantages of ACEI/ARB in patients with preserved LV systolic function. Previous meta-analysis of randomized controlled trial has revealed that ACEI/ARB only decreased cardiovascular events and death as compared to placebo and not when compared to active controls in patients with chronic coronary syndrome without heart failure^6^. Consequently, the European guideline suggested that ACEI is not generally recommended in patients with chronic coronary syndrome without heart failure. Nevertheless, they stated that ACEI should be considered in patients with significant cardiovascular risk despite the inconclusive evidence^7^.

It is conceivable that the advantages of ACEI may be influenced by initial cardiovascular risk. Less benefit with ACEI may be shown in patients with well-controlled risk factors. Currently, it is recommended that aggressive risk factor control should be implemented in patients with high cardiovascular risk^7^. As a result, the benefit of ACEI/ARB is unclear in patients with contemporary management even with the high cardiovascular risk. We aimed to investigate the effects of ACEI/ARB in patients with high atherosclerotic risk on the long-term major cardiovascular outcomes. The possible diverse effects of ACEI/ARB in different subgroup population were also explored.

## Methods

### Study population

The cohort of patients with high Risk for cardiovascular Events (CORE-Thailand) registry is a prospective, multicenter, observational, longitudinal study of Thai patients with high atherosclerotic risk. Internists, cardiologists, neurologists, endocrinologists, nephrologists, and vascular surgeons participated in the registry as investigators. There were 25 participating sites, representing diverse levels of healthcare, including 13 university-affiliated hospitals, three teaching hospitals, and nine secondary-care hospitals. These hospitals are located across the whole of Thailand in various areas. and followed at the discretion of their primary physicians. The study was approved by the Joint Research Ethics Committee and Ministry of Public Health, Thailand. Informed consent was obtained from all patients prior to the commencement of the study. The investigation conforms to the principles outlined in the Declaration of Helsinki. The study was registered at Thai Clinical Trials Registry (TCTR) https://www.thaiclinicaltrials.org/, the identification number is TCTR20130520001. The detail of the CORE-Thailand registry has been published^8^. We consecutively enrolled patients aged ≥ 45 years with established coronary artery disease (CAD), stroke /transient ischemic attack (TIA), or peripheral arterial disease (PAD), or patients with multiple risk factors (MRFs) from the outpatient clinics from April 2011 to March 2014.

The MRFs was defined as the presence of at least three atherosclerosis risk factors, including male > 55 years, female > 65 years, DM or impaired fasting glucose, hypertension, dyslipidemia, chronic kidney disease (proteinuria + 1 or eGFR < 60 ml/min) and family history of premature atherosclerotic cardiovascular diseases. Documented CAD consisted of one or more of the following criteria: stable angina with documented CAD; history of unstable angina with documented CAD; history of percutaneous coronary intervention; history of coronary artery bypass graft surgery; or previous myocardial infarction. Documented PAD consisted of one or both of the following criteria: current intermittent claudication with ankle brachial index < 0.9; and a previous history of surgery or intervention (such as angioplasty, stenting, peripheral arterial bypass graft or other vascular intervention, including amputation).

Due to the well-established benefit of ACEI/ARB in patients with LV systolic dysfunction, we did not include patients with LVEF less than 50% in our study.

### Data collection

Baseline demographic data, cardiovascular risk factors, co-morbidities, medications and laboratory data were obtained at the time of enrollment, 6 months after enrollment, and then annually for 5 years. A standardized case report form was employed to collect data locally. For individuals lost to follow-up, telephonic communication was initiated to inquire about any clinical events. In cases where telephone follow-up proved unsuccessful, the vital status was ascertained through retrieval from hospital records or the Thai Death Registration System, particularly for those patients who had provided explicit consent for data de-identification in the follow-up process. The data administration team of the Consortium of Thai Medical Schools received the patient data and transferred it to the medical research network (MedResNet). Data was checked for quality and completeness prior to data analysis. Random site monitoring was performed annually.

### The study endpoints

All participants were followed for the first occurrence of MACEs until March 2019. The primary endpoint was the effect of ACEI/ARB on long-term MACEs which were defined as a composite outcome of nonfatal myocardial infarction, nonfatal stroke, heart failure hospitalization, and death from any causes (4P-MACEs). The secondary outcome was the effect of ACEI/ARB on fatal and non-fatal myocardial infarction, fatal and non-fatal stroke, heart failure hospitalization and all-cause death. We also determined to explore the differential effect of ACEI/ARB in various subgroups.

Myocardial infarction was defined as a clinical presentation consistent with myocardial infarction accompanied by an elevation of cardiac troponin. Stroke was defined as an acute episode of focal or global neurological deficit lasting longer than 24 h, resulting from infarction or neurological deficit as a result of hemorrhage. Heart failure hospitalization was defined as hospitalization due to symptoms and signs of left or right ventricular failure.

This study was prospective in nature, and it's noteworthy that certain patients died either at home or in community hospitals. In these cases, ascertaining the precise cause of death was not always straightforward. Consequently, we chose to use all-cause mortality rather than cardiovascular mortality as the outcome in our study.

The outcomes were determined by reviewing the medical records of patients by the investigators who were not involved in the statistical analysis of the data. Subsequently, the outcomes were validated by clinicians who were not part of the study.

### Statistical analysis

Continuous variables were expressed as means and standard deviations when normally distributed, or medians and interquartile ranges when not normally distributed. The comparison across the different groups was performed using the Mann–Whitney U test or the Student t-test. Categorical variables were presented as frequency (%) and compared between groups using Fisher's exact test. The propensity score matching approach was implemented to address the balance between patients taking ACEI/ARB and those not taking them. In this process, we considered twenty-two variables, including age, sex, body mass index (BMI), systolic blood pressure (SBP), diastolic blood pressure (DBP), eGFR, DM, hypertension, dyslipidemia, current smoking, family history of atherosclerosis, metabolic syndrome, CAD, stroke or TIA, PAD, atrial fibrillation (AF), history of ventricular tachycardia (VT) or sudden cardiac arrest (SCA), and the use of antiplatelet, beta-blocker, calcium channel blocker (CCB), statin, or diuretics. No data were missing for the variables used in the propensity-score matching. Following matching, we evaluated the balance between the two groups by computing the standardized difference (Std diff). Variables with a Std diff value above 0.03 were identified as imbalanced^9^, and these were subsequently included for adjustment after propensity score matching. Cox proportional hazards models were used to estimate propensity score matching hazard ratio (HR) of MACEs. The Cox proportional hazards model was used to assess the independent effect of ACEI/ARB on the occurrence of MACEs with adjustment of residual variables with Std diff > 0.3. Statistical significance was defined as a *p* value of less than 0.05. The statistical software package SPSS version 23 (IBM Corp., Armonk, NY, USA, https://www.ibm.com/products/spss-statistics) was used for statistical analysis and STATA version 16.2 (StataCorp, College Station, TX, USA, https://www.stata.com/order/new/edu/profplus/campus-profplus/) was used for graphic creation.

### Ethics approval and consent to participate

This study was approved by the Joint Research Ethics Committee and Ministry of Public Health, Thailand (Certificate Number COA-JREC 004/2011). Informed consent was obtained from all patients prior to the commencement of the study and was registered in thaiclinicaltrials.org, identification number TCTR20130520001. The investigations were carried out following the Declaration of Helsinki, including written informed consent from all participants.

## Results

### The studied population

A total of 9390 patients were enrolled in CORE-Thailand registry. After exclusion of patients with LVEF < 50%, there were 8513 patients remained in the cohort. Of those, 5465 (64%) patients taking ACEI/ARB. The age was not different between ACEI/ARB users and non-users. However, males were more predominant in patients not taking ACEI/ARB than those taking ACEI/ARB (55.5% vs. 52.0%, *P* = 0.002). The body mass index (BMI), eGFR, systolic and diastolic blood pressure was higher in patients with ACEI/ARB therapy than those without. The patients with ACEI/ARB treatment were more likely to have DM, metabolic syndrome, hypertension and dyslipidemia. On the contrary, the patients without ACEI/ARB therapy had higher prevalence of CKD, stroke/TIA, PAD and history of VT than ACEI/ARB users. The patients taking ACEI/ARB had a greater use of antiplatelet, beta-blocker and statin.

**Figure 1:**
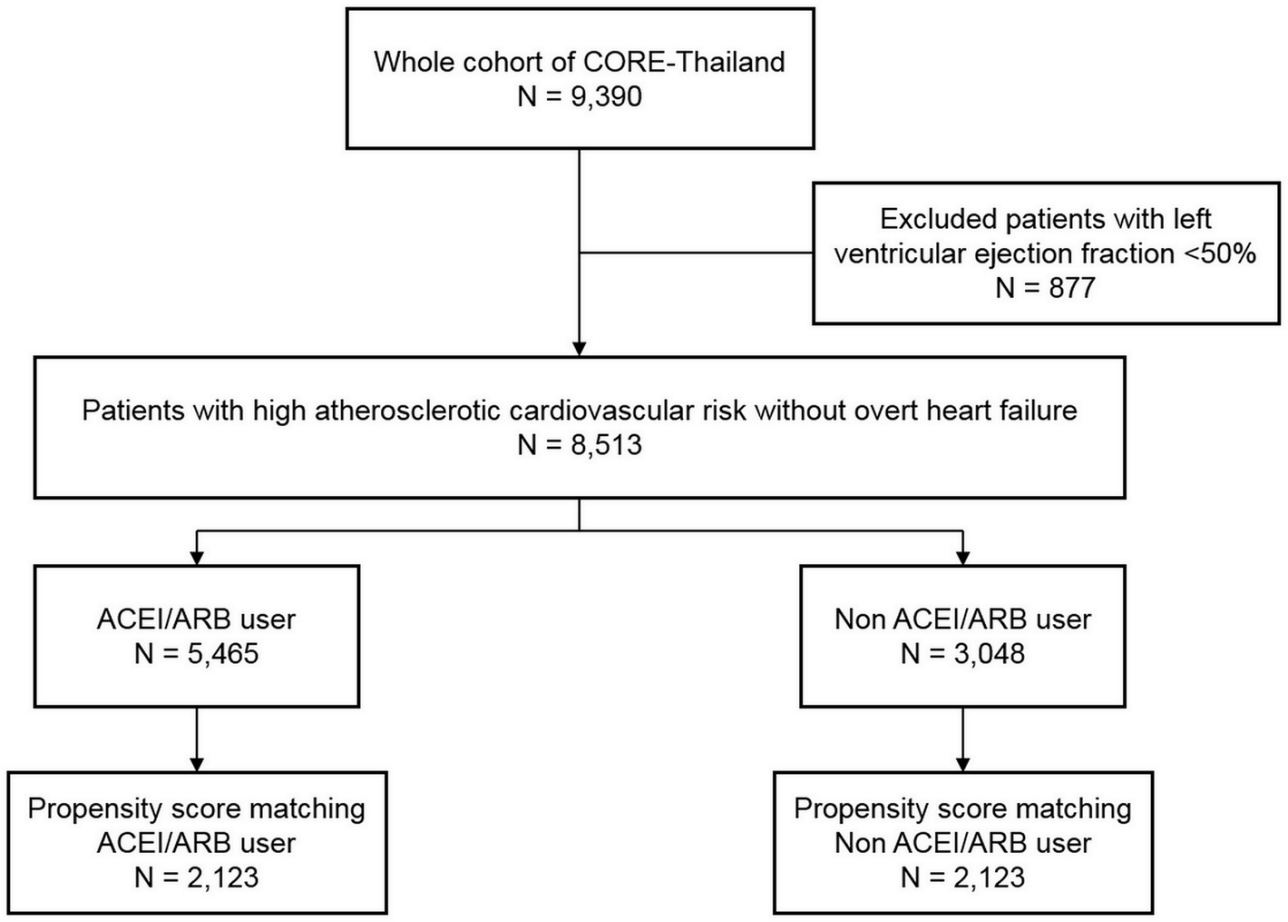
The study flow diagram.

After propensity score matching, baseline characteristics, co-morbidities, and medications were more balanced between those with and without ACEI/ARB therapy in a post-matched cohort of 4246 patients. BMI, current smoking, and chronic kidney disease were the three variables that remained imbalanced between the two groups with Std diff > 0.03. Baseline demographic data, co-morbidities and medications between ACEI/ARB users and non-users of the full cohort and after propensity score matching of the study groups are presented in Table 1. The study flow diagram of the studied population is displayed in Fig. 1.

**Table 1:** Baseline characteristics in full cohort and propensity score matching cohort.

	Full cohort					Propensity score matching cohort				
	Total	No ACEI/ARB	ACEI/ARB	*p* value	Std diff	Total	No ACEI/ARB	ACEI/ARB	*p* value	Std diff
	N = 8513	N = 3048	N = 5465			N = 4246	N = 2123	N = 2123		
Age (years)	65.7 ± 9.6	65.9 ± 9.9	65.6 ± 9.5	0.140	0.033	66.4 ± 9.7	66.4 ± 9.8	66.3 ± 9.7	0.600	0.016
Male	4534 (53.3%)	1692 (55.5%)	2842 (52.0%)	0.002	0.070	2384 (56.1%)	1190 (56.1%)	1194 (56.2%)	0.900	0.004
BMI (kg/m^2^)	25.5 ± 4.4	24.6 ± 4.1	25.9 ± 4.4	< 0.001	− 0.310	25.1 ± 4.1	25.0 ± 4.0	25.1 ± 4.1	0.240	− 0.036
eGFR (mL/min/1.73 m^2^)	67.1 ± 26.5	65.1 ± 29.5	68.1 ± 24.6	< 0.001	− 0.109	64.5 ± 27.1	64.1 ± 29.3	64.9 ± 24.8	0.340	− 0.029
SBP (mmHg)	133.3 ± 18.2	131.6 ± 18.1	134.2 ± 18.2	< 0.001	− 0.144	132.4 ± 17.9	132.4 ± 17.6	132.4 ± 18.3	0.980	< 0.001
DBP (mmHg)	74.8 ± 11.0	74.1 ± 10.6	75.1 ± 11.1	< 0.001	− 0.096	74.0 ± 10.9	74.0 ± 10.8	73.9 ± 11.1	0.770	0.009
Current smoking	433 (5.1%)	193 (6.3%)	240 (4.4%)	< 0.001	0.086	214 (5.0%)	111 (5.2%)	103 (4.9%)	0.570	0.172
Family history of atherosclerosis	677 (8.0%)	322 (10.6%)	355 (6.5%)	< 0.001	0.146	364 (8.6%)	180 (8.5%)	184 (8.7%)	0.830	0.007
Medical history										
Diabetes mellitus	5050 (59.3%)	1573 (51.6%)	3477 (63.6%)	< 0.001	0.245	2296 (54.1%)	1144 (53.9%)	1152 (54.3%)	0.810	0.008
Metabolic syndrome	4603 (54.1%)	1394 (45.7%)	3209 (58.7%)	< 0.001	0.262	2310 (54.4%)	1142 (53.8%)	1168 (55.0%)	0.420	0.025
Hypertension	8131 (95.5%)	2666 (87.5%)	5465 (100.0%)	< 0.001	0.535	4246 (100%)	2123 (100%)	2123 (100%)	NA	NA
Dyslipidemia	7405 (87.0%)	2558 (83.9%)	4847 (88.7%)	< 0.001	0.139	3666 (86.3%)	1823 (85.9%)	1843 (86.8%)	0.370	0.027
Chronic kidney disease	1794 (21.1%)	738 (24.2%)	1056 (19.3%)	< 0.001	0.119	1066 (25.1%)	576 (27.1%)	490 (23.1%)	0.002	0.094
Coronary artery disease	3374 (39.6%)	1196 (39.2%)	2178 (39.9%)	0.580	0.123	1810 (42.6%)	905 (42.6%)	905 (42.6%)	1.000	< 0.001
Stroke/TIA	778 (9.1%)	370 (12.1%)	408 (7.5%)	< 0.001	0.158	402 (9.5%)	198 (9.3%)	204 (9.6%)	0.750	0.010
PAD	235 (2.8%)	169 (5.5%)	66 (1.2%)	< 0.001	0.242	106 (2.5%)	52 (2.4%)	54(2.5%)	0.840	0.006
Atrial fibrillation	305 (3.6%)	124 (4.1%)	181 (3.3%)	0.072	0.040	171 (4.0%)	84 (4.0%)	87 (4.1%)	0.810	0.007
History of VT/SCA	123 (1.4%)	55 (1.8%)	68 (1.2%)	0.038	0.046	72 (1.7%)	39 (1.8%)	33 (1.6%)	0.480	0.023
Medication										
Antiplatelet	5813 (68.3%)	2009 (65.9%)	3804 (69.6%)	< 0.001	0.079	2888 (68.0%)	1444 (68.0%)	1444 (68.0%)	1.000	< 0.001
Beta-blocker	4418 (51.9%)	1545 (50.7%)	2873 (52.6%)	0.096	0.038	2442 (57.5%)	1225 (57.7%)	1217 (57.3%)	0.800	0.008
Calcium channel blocker	3642 (42.8%)	1295 (42.5%)	2347 (42.9%)	0.680	0.009	2069 (48.7%)	1043 (49.1%)	1026 (48.3%)	0.600	0.016
Statin	7425 (87.2%)	2543 (83.4%)	4882 (89.3%)	< 0.001	0.173	3678 (86.6%)	1843 (86.8%)	1835 (86.4%)	0.720	0.011

*BMI* body mass index, *DBP* diastolic blood pressure, *eGFR* estimated glomerular filtration rate, *NA* not applicable, *PAD* peripheral artery disease, *SBP* systolic blood pressure, *TIA* transient ischemic attack, *VT/SCA* ventricular tachycardia/sudden cardiac arrest.

### The effect of ACEI/ARB on long-term outcomes

**Figure 2:**
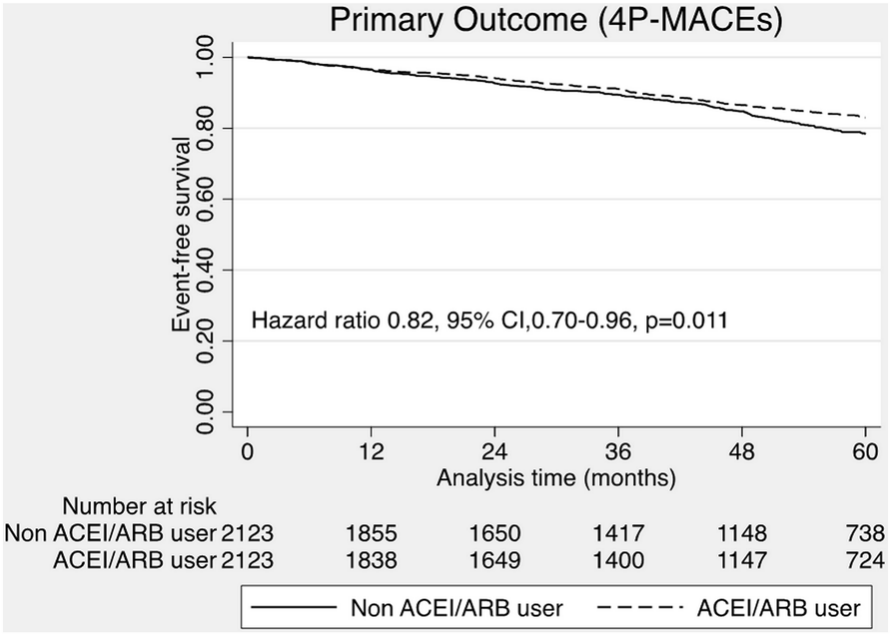
The effect of ACEI/ARB on the primary outcome (4P-MACEs).

**Figure 3:**
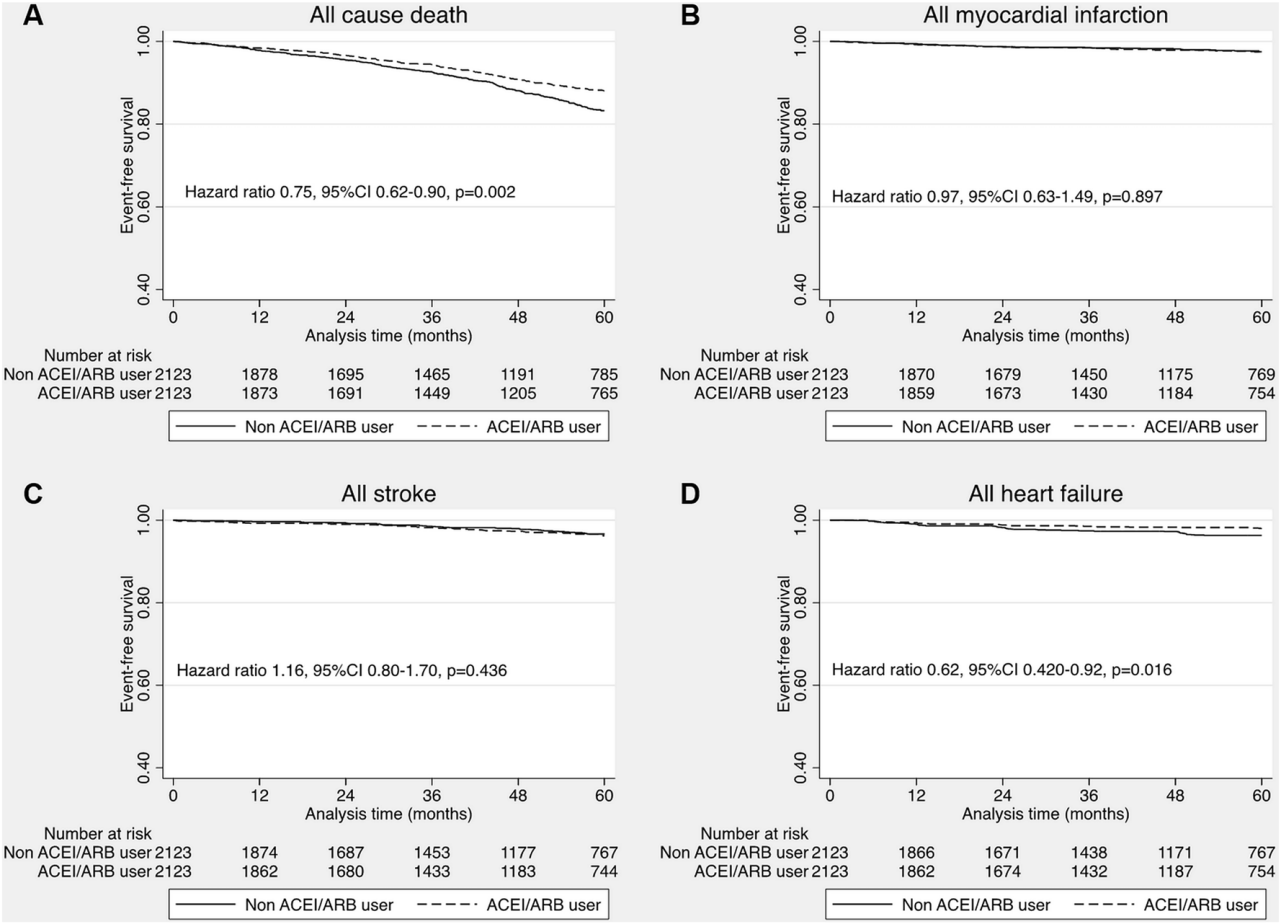
The effect of ACEI/ARB on the secondary outcomes.

Among 4246 patients in post-matching cohort, the incidence of 4P-MACEs was 15.4% during the median follow-up period of 49.6 months (IQR 24.8–60.9). There was 1.8% non-fatal myocardial infarction, 2.2% non-fatal stroke, 2.2% heart failure hospitalization and 11.1% all-cause mortality occurred during follow up. We demonstrated that the use of ACEI/ARB was significantly associated with reduced risk of 4P-MACEs (HR 0.82, 95% CI 0.70–0.96, *P* = 0.011) (Fig. 2). Double adjustment with residual confounders confirmed a lower risk of 4P-MACEs in the ACEI/ARB group (adjusted HR 0.82, 95% CI (0.71–0.96), *P* = 0.014). According to the secondary outcomes, the incidence of fatal and non-fatal myocardial infarction was 2.0%, fatal and non-fatal stroke, 2.5%, all heart failure hospitalization, 2.5% and all-cause death, 11.1%. ACEI/ARB was associated with the lower risk of all-cause mortality (HR 0.75, 95% CI 0.62–0.90, *P* = 0.002) and the lower risk of heart failure hospitalization (HR 0.62, 95% CI 0.420–0.92, *P* = 0.016). Nevertheless, the incidence of MI was not different between patients with and without ACEI/ARB therapy (HR 0.97, 95% CI 0.63–1.49, *P* = 0.897). Furthermore, the use of ACEI/ARB did not reduce the risk of stroke/TIA compared to those without ACEI/ARB therapy (HR 1.16, 95% CI 0.80–1.70, *P* = 0.436). (Fig. 3).

The differential effects of ACEi and ARB were analyzed for primary and secondary outcomes, revealing variations in outcomes between ACEi and ARB. ACEi exhibited a significant reduction in the risk of 4P-MACEs and all-cause death, consistent with the primary analysis of ACEi or ARB. In contrast, taking ARB did not result in a reduction in the incidence of 4P-MACEs or other composite outcomes (Table S1).

### The subgroup analysis on various population

**Figure 4:**
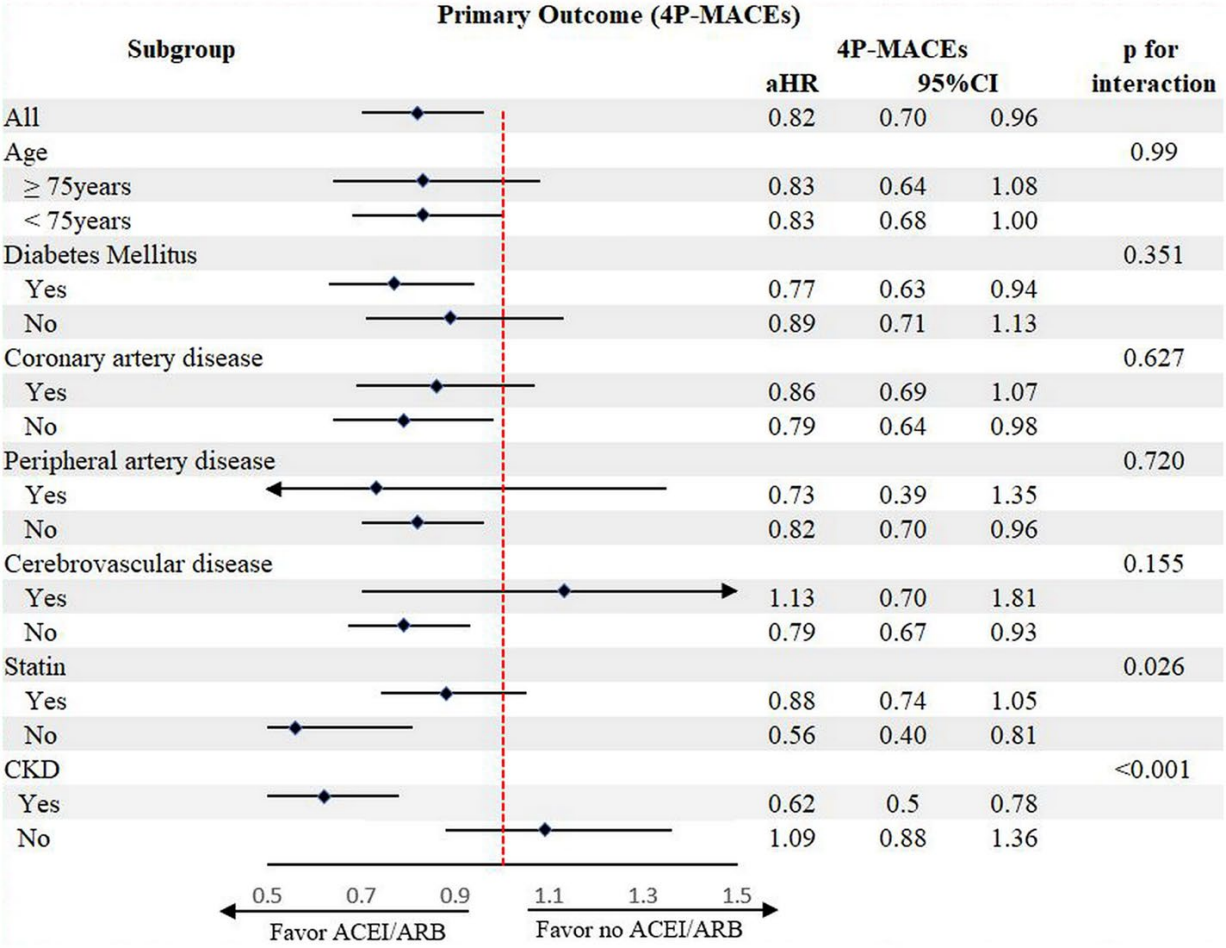
Subgroup analysis of primary outcome.

The subgroup analysis demonstrated that ACEI/ARB reduced risk of long-term 4P-MACEs in patients across the various subgroups of age 75 years or above, DM, CAD, PAD and cerebrovascular disease. Interestingly, the greater benefit of ACEI/ARB was noted in patients not taking statin (HR 0.57, 95% CI 0.40–0.82) as compared to those taking statin (HR 0.88, 95% CI 0.74–1.05, P for interaction 0.026). In addition, patients with CKD had greater benefit from ACEI/ARB (HR 0.62, 95% CI 0.50–0.78) as compared to those without CKD (HR 1.09, 95% CI 0.88–1.36, *P* for interaction < 0.001 (Fig. 4).

## Discussion

The activation of RAS primarily promotes atherosclerosis through the exertion on blood vessels via the increased oxidative stress and inflammation leading to endothelial dysfunction. Besides, the RAS activation facilitates the onset of diabetes, obesity, insulin resistance and hypertension which are the robust risk factor for atherosclerosis^10,11^. Therefore, RAS blockers have a potential to decrease atherosclerotic plaque progression and ischemic events. However, the European guideline^7^ stated that ACEI is not generally recommended in patients with coronary artery disease without overt heart failure. This recommendation is based on several randomized trials of ACEIs that did not show the benefit of ACEIs over placebo in patients with chronic coronary syndrome. These studies included PEACE (Prevention of Events with Angiotensin Converting Enzyme Inhibition)^12^, QUIET (Quinapril Ischemic Event Trial)^13^ and IMAGINE (Ischemia Management With Accupril Post-Bypass Graft via Inhibition of the Converting Enzyme)^14^.

Nevertheless, they suggested that ACEI be taken into account in individuals with significant cardiovascular risk according to the results of HOPE and EUROPA studies^15,16^. The HOPE study (Heart Outcomes Prevention Evaluation) demonstrated that ramipril significantly reduced MACEs in patients with high atherosclerotic cardiovascular risk and preserved LV systolic function^16^. Similarly, EUROPA study (EUropean trial on Reduction Of cardiac events with Perindopril in patients with stable coronary Artery disease) showed that perindopril decreased the risk of MACEs in patients with chronic coronary syndrome without impaired LV systolic function^15^.

The potential explanations have been proposed for the discrepancy in the results including different ACEI or dose used, the different study design and the different baseline risk population. The patients in PEACE trial had higher rate of coronary revascularization than those in HOPE and EUROPA trials. The IMAGINE trial included the patients with recent coronary artery bypass grafting and excluded patients with diabetes mellitus. The enrolled subjects in these trials represented the relatively low risk population. As a result, the placebo groups in PEACE, QUIET and IMAGINE had lower annualized rates of cardiovascular death and nonfatal MI compared to those in HOPE trial. In addition, the QUIET trial has been criticized for the insufficient statistical power to detect hard outcomes and the relatively short follow-up period of the study.

In agreement with HOPE and EUROPA studies, we demonstrated that the use of ACEI was associated with the lower risk of long-term MACEs in patients with high atherosclerotic cardiovascular risk. Likewise, several observational studies have shown that RAS blockers were associated with the lower risk of MACEs in patients with chronic coronary syndrome^17–19^.

Nevertheless, the benefit of ACEI/ARB observed in our study was driven significantly from the reduction in heart failure hospitalization and all-cause mortality. The incidences of MI and stroke/TIA were not different between patients with and without ACEI/ARB therapy. Our results were in contrast with those reported in HOPE and EUROPA trials which showed that ramipril and perindopril reduced the risk of MI and stroke^15,16^. In subgroup analysis, we revealed that patients not taking statin were likely to benefit in a greater extent than those taking statin. The discrepancy in the results may be explained by the difference in the rate of statin use. In our studied population, the rate of statin use was 87% which was much higher than 29% of lipid lowering therapy reported in HOPE trial and 57% reported in EUROPA trial^15,16^. Accordingly, the patients with well-controlled risk factors may benefit less from ACEI/ARB therapy in terms of the reduction in atherosclerotic events.

Heart failure is an important adverse event in high atherosclerotic risk populations; therefore, heart failure hospitalization has been included in the combined outcomes of studies involving these patients. However, previous ACEI trials in this population did not incorporate heart failure as a primary outcome, except for IMAGINE trial^14^. Nevertheless, the IMAGINE trials did not include individuals with DM, a significant risk factor for heart failure that may benefit from RAS inhibition^20^. The studies evaluating effects of ARB in high cardiovascular risk patients, such as ONTARGET (ONgoing Telmisartan Alone and in combination with Ramipril Global Endpoint Trial)^21^ and TRANSCEND (Telmisartan Randomised AssessmeNt Study in ACE iNtolerant subjects with cardiovascular Disease)^22^ included heart failure event in their primary outcomes. The ONTARGET study demonstrated equivalent effects of ACEi and ARB, but there was a neutral effect of ARB compared to placebo in patients intolerant to ACEi. When heart failure hospitalization was excluded from the primary outcome, and outcomes used in the HOPE study were considered, a modest benefit of ARB was demonstrated^22^. The controversy surrounding the differential effects of ACEi and ARB stems from differences in the studied populations, concomitant treatments such as statins or antiplatelets, as well as comparators. Although divergent effects of ACEIs and ARBs were observed in post hoc analyses and our analysis, the head-to-head comparison in the ONTARGET trial did not show a significant difference between ACEi and ARB^21^. The effects of ACEi and ARB may vary among patient groups, and further study is warranted.

The present study had some limitations. The results of this prospective, observational study may be affected by unmeasured confounders and residual confounding, even if propensity score matching was used to account for known confounders. The information about prior ACEI/ARB use, indication, and medication compliance was not evaluated. The phenotype of heart failure event was not classified. Due to the nature of a cohort study, the non-fatal clinical events may be underreporting which lead to the lower incidence of non-fatal myocardial infarction or non-fatal stroke. However, the similar pattern showing lower incidence of non-fatal myocardial infarction and non-fatal stroke was also presented in the observational study^23^ or randomized control trial in patients with high atherosclerotic risk^21,24^.

## Conclusion

In a large cohort of high atherosclerotic risk patients without overt heart failure, we demonstrated that the use of ACEI/ARB was associated with the reduced risk of long-term MACEs. The reduction of all-cause mortality and heart failure hospitalization was the main contributor to the benefit of ACEI/ARB therapy. Further investigations may be needed to explore potential differential effects between ACEI and ARB.

### Supplementary Information

Supplementary Table S1.

## Data Availability

The data that support the findings of this study are available from the corresponding author upon reasonable request.
