# Supplementary material for: Effect of renin angiotensin system inhibitors on long-term major cardiovascular outcomes in patients with high atherosclerotic cardiovascular risk
Source: Sci Rep. 2023 Dec 27;13:23066. doi: 10.1038/s41598-023-50430-8 (PMC10754885; doi:10.1038/s41598-023-50430-8)
Supplement: Supplementary file 1 — Supplementary Table S1. [file 41598_2023_50430_MOESM1_ESM.docx]

**Table S1.** Differential effects of ACEI and ARB on primary and secondary outcomes

|  | **ACEI** | | **ARB** | |
| --- | --- | --- | --- | --- |
|  | **HR (95%CI)** | **p-value** | **HR (95%CI)** | **p-value** |
| 4P-MACES | 0.82 (0.68-0.99) | 0.039 | 0.95 (0.80-1.13) | 0.60 |
| All-cause death | 0.79 (0.63-0.99) | 0.042 | 0.89 (0.73-1.10) | 0.296 |
| All-MI | 1.12 (0.75-1.92) | 0.457 | 0.80 (0.48-1.33) | 0.385 |
| All-stroke | 0.75 (0.46-1.21) | 0.233 | 1.49 (1.00-2.20) | 0.049 |
| All heart failure | 0.75 (0.47-1.21) | 0.242 | 0.63 (0.39-1.02) | 0.062 |
